# Long noncoding RNA PVT1 inhibits renal cancer cell apoptosis by up-regulating Mcl-1

**DOI:** 10.18632/oncotarget.21706

**Published:** 2017-10-09

**Authors:** Qingjian Wu, Fan Yang, Zhenxing Yang, Zhenqiang Fang, Wanlei Fu, Wei Chen, Xiaobing Liu, Jiang Zhao, Qingqing Wang, Xiaoyan Hu, Longkun Li

**Affiliations:** ^1^ Department of Urology, Second Affiliated Hospital, Third Military Medical University, Chongqing 400037, China; ^2^ Center of Medical Experiment & Technique, Second Affiliated Hospital, Third Military Medical University, Chongqing 400037, China; ^3^ Department of Pathology, Second Affiliated Hospital, Third Military Medical University, Chongqing 400037, China

**Keywords:** plasmacytoma variant translocation 1 (PVT1), clear cell renal cell carcinoma (CCRCC), long noncoding RNA (lncRNA), apoptosis

## Abstract

Long non-coding RNA plasmacytoma variant translocation 1 (PVT1) is up-regulated in various human cancers, and our results indicated that PVT1 was up-regulated in clear cell renal cell carcinoma tissues. The Cancer Genome Atlas cohort analysis revealed that in clear cell renal cell carcinoma, higher PVT1 expression correlated with advanced TNM stage, histological grade, and poor survival. PVT1 knockdown promoted apoptosis, inhibited renal cancer cell proliferation, decreased Mcl-1, and increased cleaved caspase-3 and cleaved PARP. PVT1 increased Mcl-1 mRNA levels in renal cancer cells by promoting mRNA stability without influencing its transcription. *in vitro*, the enhanced apoptosis arising from PVT1 suppression was attenuated by overexpressing Mcl-1. In addition, *in vivo* experiments showed that PVT1 knockdown repressed xenograft tumor growth, while Mcl-1 overexpression partially rescued xenograft tumor growth. These results indicate the PVT1/Mcl-1 pathway inhibits renal cancer cell apoptosis *in vitro* and *in vivo*. PVT1 may thus serve as a novel biomarker, and the PVT1/Mcl-1 pathway may be a useful therapeutic target for clear cell renal cell carcinoma.

## INTRODUCTION

Renal cell carcinoma (RCC) is the most common malignant neoplasm of the kidney, which accounts for 2-3% of all adult malignancies [1]. The incidence and mortality rate of RCC has increased in recent years, especially in young patients and those with high-grade disease [2, 3]. RCC incidence in China has risen rapidly in populations >35 years of age, and there were 45,096 new RCC cases in China in 2011 [4]. Due to the lack of early detection and RCC prognostic markers, 25%-30% of patients have already developed metastases at the time of diagnosis. Radical nephrectomy is the principal and most effective treatment for RCC, but more than 40% of patients develop metastases after radical nephrectomy with poor prognosis [5]. Clear cell renal cell carcinoma (CCRCC) is the most common and most aggressive histologic subtype of RCC, which accounts for 75%–80% of RCC [6]. We investigated the molecular mechanisms of CCRCC formation and development to identify reliable biomarkers and novel therapeutic targets for CCRCC.

Long noncoding RNAs (lncRNAs) are a class of RNA with transcripts longer than 200 nucleotides that lack functional open reading frames [7]. Many lncRNAs are involved in tumorigenesis [8], and can act as tumor suppressors or oncogenes in cancers and may be developed as useful, noninvasive, diagnostic and prognostic biomarkers across a series of cancer types [9]. Long noncoding RNA Plasmacytoma variant translocation 1 (PVT1) locates at the human chromosome 8q24, a region with frequent copy number amplifications [10]. PVT1 is overexpressed, inhibits apoptosis, and is associated with tumorigenesis and poor prognosis in many cancers [11–16].

## RESULTS

### PVT1 was up-regulated in CCRCC tissues and was tightly associated with clinical significance of CCRCC

**Figure 1:**
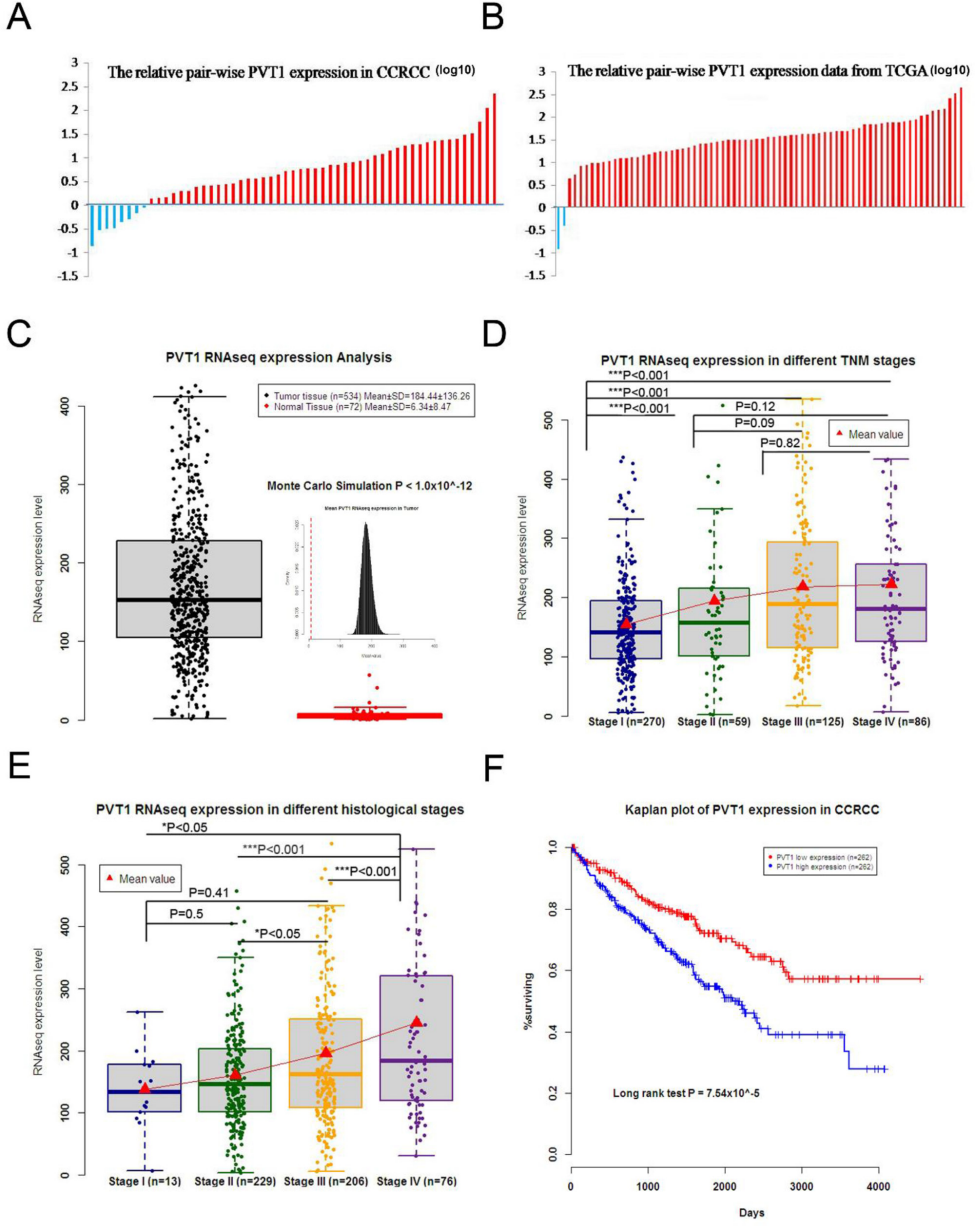
PVT1 was up-regulated in CCRCC, which was also associated with the clinical significance of CCRCC **(A)** PVT1 levels were higher in most of the CCRCC tissues than the adjacent non-tumor tissues of 55 pair-wise samples. **(B)** TCGA data of 72 pair-wise samples denoted that 70 of 72 show higher expression in CCRCC tissues than adjacent non-tumor tissues. **(C)** TCGA data implicated that PVT1expression was up-regulated in CCRCC tissue compared to normal tissue, and higher PVT1expression was also correlated with advanced TNM stage **(D)** and histological grade **(E)**. **(F)** Kaplan–Meier analysis of overall survival was analyzed according to PVT1 levels from TCGA. (^*^, P < 0.05; ^**^, P < 0.01; ^***^, P < 0.001).

Compared with corresponding adjacent non-tumor tissues, PVT1 was up-regulated in 85.45% (47 of 55) of CCRCC tissues according to qRT-PCR (Figure 1A). This finding was lower than the data from TCGA (97.2%) (Figure 1B), but not significantly different. Data from TCGA demonstrated that PVT1 expression was up-regulated in CCRCC tissues compared to normal tissues (534 tumor tissues and 72 adjacent non-tumor tissues) (Figure 1C). TCGA data showed that higher PVT1 expression was correlated with advanced TNM stage (Figure 1D), histological grade (Figure 1E), and poor survival (Figure 1F). These results indicate that PVT1 might be tumorigenic and stimulate the development and progression of CCRCC.

### PVT1 knockdown inhibited renal cancer cell proliferation and colony formation *in vitro*

**Figure 2:**
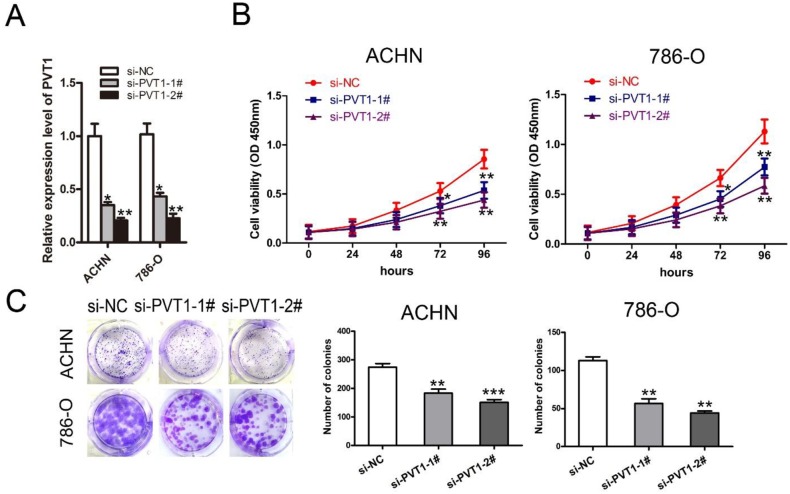
Knockdown of PVT1 inhibited renal cancer cell proliferation and colony formation *in vitro* **(A)** The qRT-PCR analysis of PVT1 expression in 786-O and ACHN cells transfected with si-NC, si-PVT1-1, or si-PVT1-2. **(B)** The cell proliferation of ACHN and 786-O cells transfected with si-NC, si-PVT1-1, or si-PVT1-2 was detected by the CCK-8 assay. **(C)** Colony formation assay detected proliferation of ACHN and 786-O cells transfected with si-NC, si-PVT1-1, or si-PVT1-2. (^*^P < 0.05, ^**^P < 0.01, ^***^P < 0.001).

To evaluate the function of PVT1 in renal cancer cell apoptosis and proliferation, two independent siRNAs against PVT1 were transfected into renal cancer cell lines 786-O and ACHN, respectively. PVT1 expression was reduced by siPVT1-1 or si-PVT1-2, which was confirmed by qRT-PCR analysis in both cell lines (Figure 2A). CCK-8 and colony formation assays also showed that 786-O and ACHN cell proliferation and colony-forming efficiencies decreased with PVT1 knockdown (Figure 2B and 2C).

### Knockdown of PVT1 promoted renal cancer cell apoptosis by down-regulating Mcl-1

**Figure 3:**
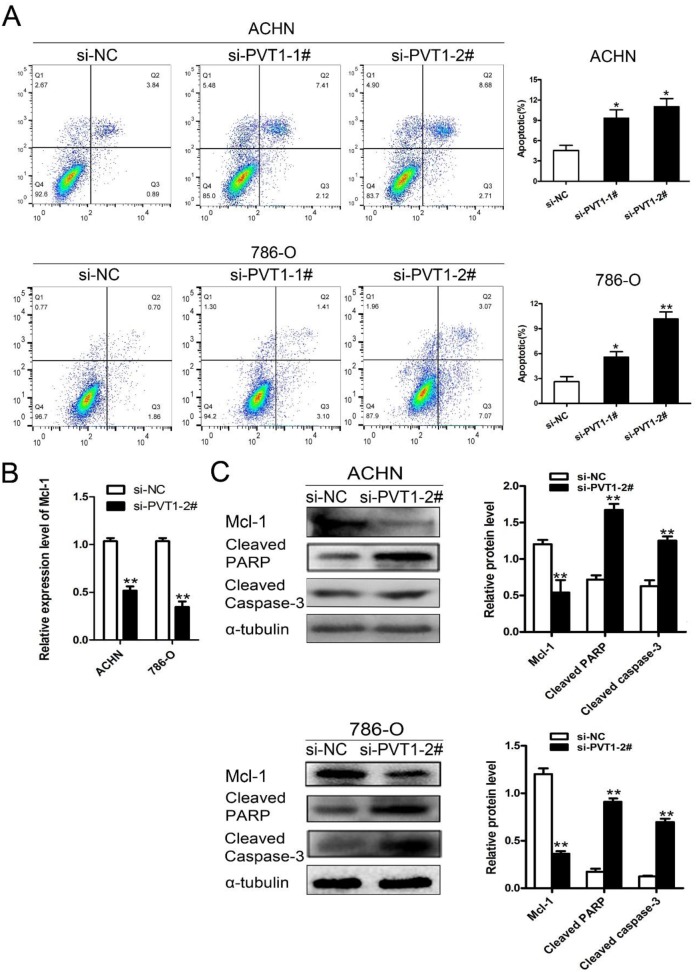
Knockdown of PVT1 promoted renal cancer cell apoptosis by down-regulating Mcl-1 **(A)** Flow cytometry analyzed the cell apoptosis of ACHN and 786-O cells transfected with si-NC, si-PVT1-1, or si-PVT1-2 for 48 h. **(B)** Mcl-1 levels in ACHN and 786-O cells transfected with si-NC or si-PVT1-2 were detected by qRT-PCR. **(C)** Western blot analysis of Mcl-1, cleaved PARP and cleaved Caspase-3 in ACHN and 768-O cells transfected with si-NC or si-PVT1-2. (^*^P < 0.05, ^**^P < 0.01).

Mcl-1 is an anti-apoptotic member of the Bcl-2 family, which regulates apoptosis through both pro-apoptotic and anti-apoptotic factors [17]. Mcl-1 overexpression is one of the most common genetic abnormities in a variety of human cancers, and can be the cause of resistance to several chemotherapeutic agents [18]. In our study, flow cytometry showed that PVT1 down-regulation promoted cell apoptosis of 786-O and ACHN cells compared with the si-NC group. (Figure 3A). Mcl-1 expression was down-regulated in the si-PVT1 group compared with si-NC group in 786-O and ACHN cells according to qRT-PCR (Figure 3B). Western blot analysis showed that Mcl-1 was down-regulated with PVT1 knockdown, and that the expression of cleaved caspase-3 and cleaved PARP were increased in 786-O and ACHN cells (Figure 3C). The results suggest that silencing PVT1 down-regulated Mcl-1 and promoted apoptosis.

### Enhanced apoptosis and suppressed colony formation from silencing PVT1 was attenuated by overexpressing Mcl-1

**Figure 4:**
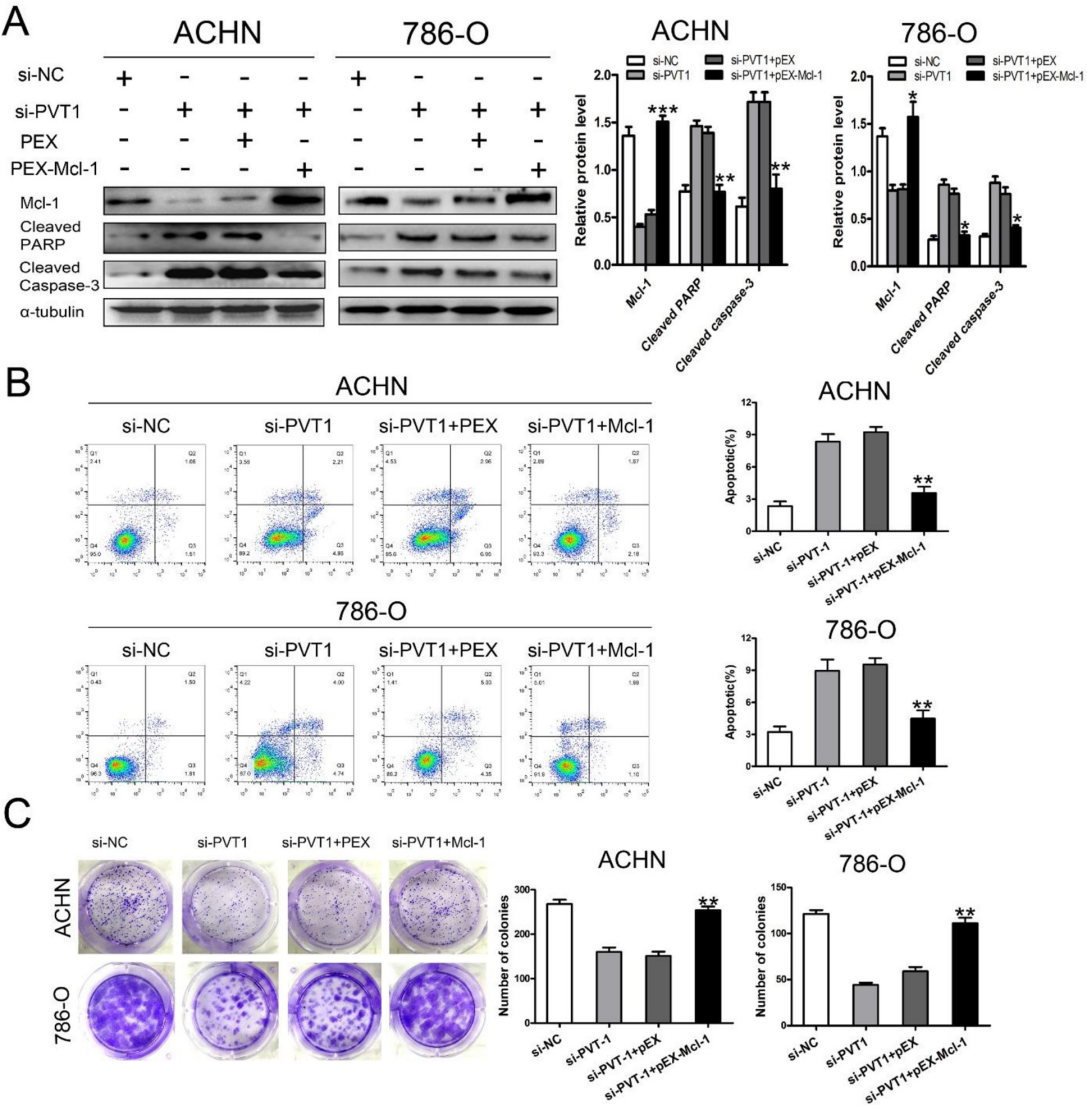
Enhanced apoptosis from silencing PVT1 were rescued by overexpressing Mcl-1 in renal cancer cells **(A)** Western blot analysis of Mcl-1, cleaved Caspase-3, and cleaved PARP in PVT1 knockdown ACHN and 786-O cells that were cotransfected with or without overexpressing Mcl-1. Apoptosis analysis **(B)** and colony formation assay **(C)** of PVT1 knockdown ACHN and 786-O cells that were cotransfected with or without overexpressing Mcl-1. (^*^p < 0.05, ^**^p < 0.01, and ^***^p < 0.001).

We next examined the contribution of Mcl-1 to the increase in cellular apoptosis after knocking down PVT1. Mcl-1 expression was up-regulated by transfecting pEX-Mcl-1, which was confirmed by the qRT-PCR in both ACHN and 786-O cells (Supplementary Figure 1). Flow cytometry confirmed that the knockdown of PVT1 enhanced apoptosis and increased cleaved caspase-3 and cleaved PARP expression. These effects were attenuated by overexpressing Mcl-1 (Figure 4A and 4B). The suppressed colony formation from knocking-down PVT1 was rescued by overexpressing Mcl-1(Figure 4C). These results revealed that the anti-apoptotic function of PVT1 was induced by Mcl-1 expression.

### PVT1 enhances the mRNA stability of Mcl-1

**Figure 5:**
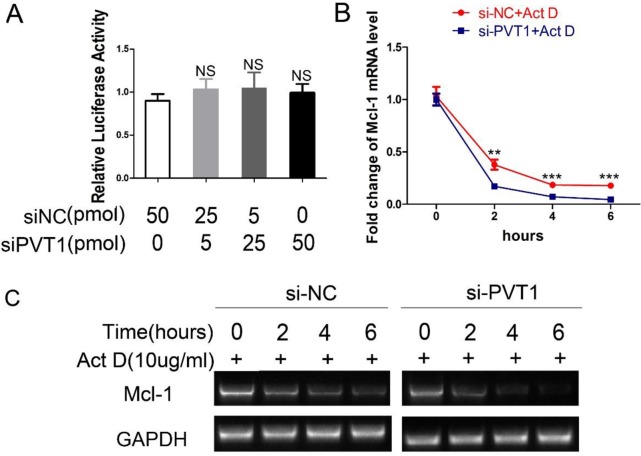
PVT1 decreases Mcl-1 mRNA stability instead of its transcriptional activity **(A)** After cotransfection with pGL3-Mcl-1 and siPVT1/siNC and pRL-TK for 48 h, the luciferase activity was assayed using the Dual-Luciferase Reporter System and normalized to the control. ACHN cells were transfected with siNC or siPVT1. After 48h, they were treated with 10ug/ml actinomycin D (Act D) for 0h/2h/4h/6h. The transcription level of Mcl-1 was assayed using qRT-PCR **(B)** and RT-PCR **(C)**. (^**^p < 0.01; ^***^P < 0.001).

To investigate the mechanisms of PVT1-mediated upregulation of Mcl-1, we first assessed whether PVT1 affected Mcl-1 at the transcriptional level. The promoter region (-1695+208) of *Mcl-1* gene was cloned into the pGL3-Basic reporter vector, which was named pGL3-Mcl-1. Luciferase reporter assay revealed that PVT1 could not affect the transcriptional activity of the *Mcl-1* gene promoter (Figure 5A). Secondly, we examined PVT1's effect on the mRNA stability of Mcl-1 in ACHN cells. When treated with transcription inhibitor actinomycin D (Act D), Mcl-1mRNA levels in ACHN cells transfected with siPVT1 were lower than those transfected with siNC (Figure 5B and 5C). This indicates PVT1 could upregulate Mcl-1 through enhancing mRNA stability in ACHN cells.

### PVT1 promoted renal cancer cell growth and inhibited apoptosis by promoting Mcl-1 in vivo

**Figure 6:**
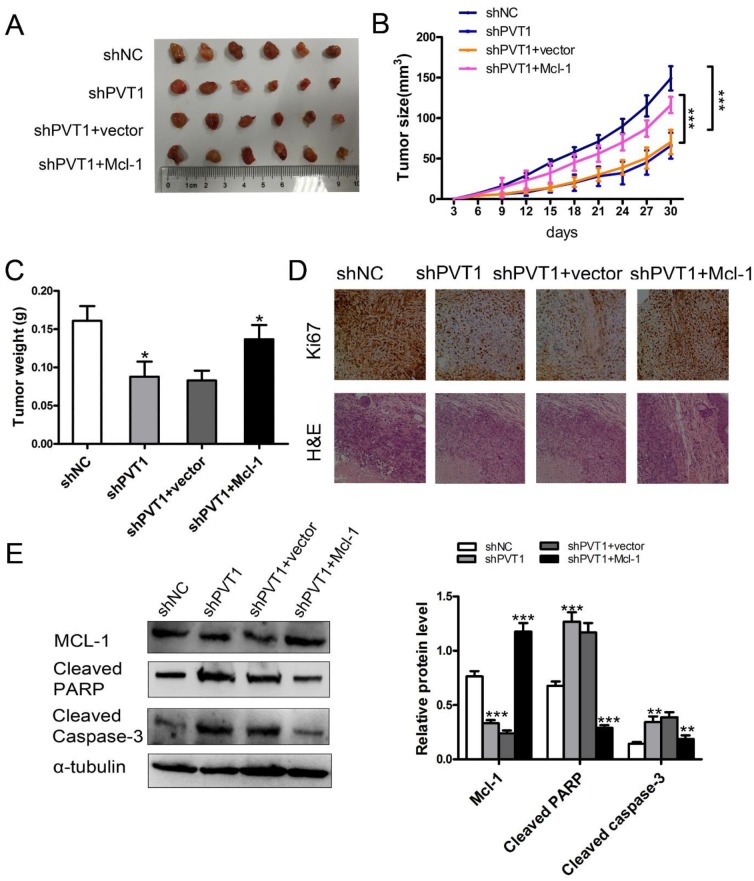
PVT1 inhibits renal cancer cell growth and apoptosis by Mcl-1 *in vivo* **(A)** Images of the xenograft tumors from ACHN cell stably transfected with sh-NC, sh-PVT1, sh-PVT1+vector, and sh-PVT1+Mcl-1 injected into the nude mice after 30 days. **(B)** Tumor volumes were calculated every three days after three days of injection. **(C)** Tumor weights are represented as means of tumor weights ± SD. **(D)** The tumor sections underwent H&E staining and IHC staining using antibodies against Ki-67. **(E)** Western blot analysis of Mcl-1, cleaved Caspase-3, and cleaved PARP in xenograft tumors from ACHN cells stably transfected with sh-NC, sh-PVT1, sh-PVT1+vector or shPVT1+ Mcl-1. (^*^p < 0.05, ^**^p < 0.01, and ^***^p < 0.001).

To evaluate the above phenomenon *in vivo*, we established xenograft tumor models in nude mice using the ACHN cell line with or without stable knockdown of PVT1 and stable Mcl-1 overexpression. We found that silencing PVT1 reduced xenograft tumor growth, which was partially rescued by Mcl-1 overexpression (Figure 6A-6C). Immunohistochemistry staining revealed that Mcl-1 overexpression alleviated Ki67 downregulation (Figure 6D). PVT1 and Mcl-1 levels in xenograft tumors were detected with qRT-PCR (Supplementary Figure 2). Western blot of cleaved PARP and cleaved Caspase-3 revealed that Mcl-1 promotes PVT1-induced renal cancer cell growth and inhibits apoptosis in vivo (Figure 6E).

## DISCUSSION

Many long non-coding RNAs have been identified in various cancer genomes, and can be used as novel biomarkers and therapeutic targets for cancer [19]. PVT1 is among those long non-coding RNAs, which is overexpressed and associated with tumorigenesis and poor prognosis in a range of cancers [11–16, 20]. TCGA cohorts revealed that among all cancer types, CCRCC showed the strongest up-regulation of PVT1, and the highest level of PVT1 correlated with poor clinical outcome [21]. Our data also showed that PVT1 was increased in CCRCC tissues versus the corresponding non-tumor tissues. PVT1 might stimulate the development and deteriorate the prognosis of CCRCC. Moreover, our study revealed that PVT1 knockdown could inhibit proliferation and induce apoptosis of renal cancer cells *in vivo* and *in vitro*.

Mcl-1 is an anti-apoptotic member of the Bcl-2 family [22], and it is up-regulated in a variety of human cancers [23] and highly expressed in many human cancer cell lines [24]. The up-regulation of Mcl-1 results in resisting cell death, increasing cell proliferation, and tumor cell survival [25]. Down-regulation of Mcl-1 has shown tumor growth inhibition in colon, lung, ovarian cancer cells and lymphoma cells by inducing apoptosis. Knockdown of Mcl-1 restores sensitivity to chemotherapy in chemoresistant cells [18].

Rather than enhancing transcriptional activity of the *Mcl-1* promoter, our data showed that PVT1 enhanced the stability of Mcl-1 mRNA in renal cancer cells. RNA stability is affected by various factors such as RNases, RNA binding proteins, miRNAs, and lncRNAs. Subramanian D reported in 2008 that RNA binding protein CUGBP2 promoted Mcl-1 mRNA stabilization [26]. Our next study will investigate whether PVT1-enhanced Mcl-1 mRNA stability is associated with CUGBP2.

MicroRNAs can suppress mRNA stability by targeting mRNA directly. Bioinformatic analysis by TargetScan and Miranda revealed putative miRNA response elements (MREs) of miR-29b-3p/ miR-30C-5p/ miR-106a-5p shared by PVT1 (Supplementary Figure 3A) and the 3′UTR of Mcl-1 [27–29]. Those three miRNAs can directly bind to 3′UTR of Mcl-1to decrease Mcl-1 mRNA stability [27–29]. We detected pmiR-RB-PVT1luciferase activity with or without miR-29b-3p/ miR-30C-5p/ miR-106a-5p mimics and inhibitors. The luciferase activity of pmiR-RB-PVT1 which contained PVT1 at the 3′UTR of Rluc showed no response to miR-29b-3p/ miR-30C-5p/ miR-106a-5p mimics and inhibitors (Supplementary Figure 3B and 3C). These data demonstrate that PVT1 does not contain functional binding sites of miR-29b-3p/ miR-30C-5p/ miR-106a-5p to support Mcl-1 mRNA stability.

The present study found that PVT1 expression was increased in CCRCC, correlated with advanced TNM stage, histological grade, and poor survival of CCRCC. We also showed that PVT1 inhibits renal cancer cell apoptosis by enhancing Mcl-1 mRNA stability. These findings suggest that PVT1 may be oncogenic and a CCRCC biomarker, and that the PVT1/Mcl-1 pathway may serve as a novel therapeutic target for treating CCRCC.

## MATERIALS AND METHODS

### Patients and specimens

From June 2015 to May 2016, 55 pairs of renal cancer specimens and the corresponding adjacent non-tumor tissues were collected from patients who had undergone radical nephrectomy at the Department of Urology, Xinqiao Hospital, The Third Military Medical University, Chongqing, P. R. China. All the patient specimens were diagnosed as CCRCC by histopathological examination. The research was approved by the ethical committee of the Third Military Medical University of China, and written informed consent was provided by each patient before surgery. Part of the PVT1 RNA-seq expression data is available from TCGA database (http://cancergenome.nih.gov/) according to TCGA publication guidelines.

### Cell culture

ACHN and 786-O cell lines were purchased from the Cell Bank of the Chinese Academy of Sciences (Shanghai, China). ACHN was cultured in MEM (Gibco, Carlsbad, CA, USA) and 786-O was cultured in RPMI-1640 (Gibco, Carlsbad, CA, USA) supplemented with 10% FBS, at 37°C in a 5% CO2 incubator.

### RNA extraction and qRT-PCR analyses

Total RNA was extracted from cells or tissues using the RNAiso Plus (Takara, Dalian, China). RNA samples were reverse transcribed with PrimeScript™ RT reagent Kit with gDNA Eraser (Takara, Dalian, China). SYBR Green PCR kit (Takara, Dalian, China) was used for the qRT-PCR, with GAPDH as the endogenous control. The primer pairs were listed as follows: 5′- TGAGAACTGTCCTTACGTGACC -3′ (Forward) and 5′- AGAGCACCAAGACTGGCTCT -3′ (Reverse) for PVT1, 5′- TCTCTCGGTACCTTCGGGAG -3′ (Forward), 5′- GTCACAATCCTGCCCCAGTT -3 (Reverse) for Mcl-1, and 5′- CATCAAGAAGGTGGTGAAGCAG -3′(Forward) and 5′-CGTCAAAGGTGGAGGAGTGG -3′ (Reverse) for GAPDH. The primers for miRNAs and U6 were purchased from Ribobio (Ribobio, Guangzhou, China).

### RNA interference and transfection

Small interfering RNA (siRNA) and nonspecific control siRNA (siNC) were synthesized (GENEPHAM, Shanghai, China) and transfected into 786-O or ACHN cells by using Lipofectamine 3000 (Invitrogen, USA), according to the manufacturer's protocol. Forty-eight hours after transfection, cells were harvested and subjected to qRT-PCR or western blot analyses. The sequences of si-PVT1 are listed as follows: si-PVT1-1#, 5′-GCUUGGAGGCUGAGGAGUUTT -3′; si-PVT1-2#, 5′-CCCAACAGGAGGACAGCUUTT-3′. The miRNA mimics and inhibitors were purchased from Ribobio (Ribobio, Guangzhou, China).

### Cell proliferation assays

Cells were seeded at a density of 2 × 10^3^ cells/ well in a 96-well plate for 24 h, then transfected with si-PVT1 or negative control. Cell proliferation was tested with CCK-8 (Dojindo, Kumamoto, Japan) according to the manufacturer's instructions. All experiments were performed at least three times.

### Colony formation assay

Cells transfected with siRNAs were plated on 6-well plates (1 × 10^3^ cells per well) and maintained in proper medium containing 10% FBS for 12 days. Colonies were fixed with methanol and stained by 0.1% crystal violet (Beyotime Institute of Biotechnology, Shanghai, China). Visible colonies were photographed and counted manually.

### Apoptosis analysis by flow cytometry

Cells were harvested 48 h after si-PVT1 or si-NC transient transfection and incubated with annexin V-FITC and PI according to the manufacturer's instructions (BD, San Diego, CA, USA). Apoptosis was analyzed by a flow cytometer (MoFlo, Beckman, CA, USA).

### Western blot assay and antibodies

Total protein was isolated from cells and tumor tissues with RIPA lysis buffer (Beyotime, Jiangsu, China). Proteins were separated by SDS-PAGE and transferred onto PVDF membranes. Membranes were blocked with 5% non-fat milk in TBST, incubated with primary antibodies at 4°C overnight, and further incubated with horseradish peroxidase-conjugated secondary antibodies for 1 h at 37°C. Proteins were visualized using ECL reagents (Thermo Scientific, Waltham, MA, USA) according to the manufacturer's instructions. Protein levels were normalized against α-tubulin. Antibodies of cleaved caspase-3, cleaved PARP, and α-tubulin were obtained from Cell Signaling Technology (CST, Danvers, MA, USA) and Mcl-1 antibodies were obtained from Santa Cruz Biotechnology (Santa Cruz, CA, USA).

### Plasmid generation

The pEX–Mcl-1 and pEX negative control vectors were purchased from Genepharma (Shanghai, China) for ectopic expression in cells. The qRT-PCR assay was conducted to evaluate Mcl-1 expression. The fragment containing human Mcl-1 promoter regions (-1693~+208) was chemically synthesized and cloned into the pGL3-Basic vector, and the resulting plasmid was named pGL3-Mcl-1. The fragment containing human PVT1 was chemically synthesized and cloned into the pmiR-RB-REPORT (Ribobio, Guangzhou, china), and the resulting plasmid was named pmiR-RB-PVT1.

### Establishment of stable cells

The shRNA-PVT1 (5′-CCCAACAGGAGGACA GCTT-3′) and control shRNA were separately inserted into the vector pGLV3/H1/GFP/Puro from Genepharma (Shanghai, China). The packaged lentivirus containing pGLV3/H1/GFP/Puro/shPVT1 or pGLV3/H1/GFP/Puro/shNC was used to infect ACHN cells at a multiplicity of infection (MOI) of 10, followed by screening with puromycin. The resulting ACHN cells with stable PVT1knockdown were named shPVT1. The control ACHN cells with stable shNC expression were named shNC. The packaged lentivirus containing pGLV5/H1/GFP/Puro/Mcl-1 and negative control from Genepharma (Shanghai, China) separately infected ACHN cells with stable shPVT1expression at MOI of 10, followed by a puromycin screening. These two subgroup cells were named shPVT1+Mcl-1 and shPVT1+vector.

### Luciferase reporter assay

ACHN cells were seeded in 24-well plates and grown to 70–80% confluence, and 12hours later they were transiently transfected using Lipofectamine3000 (Invitrogen). The cells were cotransfected with pGL3-Mcl-1 and monitor plasmid pRL-TK (Promega, Madison, USA). After 48 h, luciferase activity was measured using the Dual-Luciferase Reporter Assay System (Promega, Madison, USA). Data are represented as the fold induction after normalizing the luciferase activity of the tested sample to that of the corresponding control sample. The transfection experiments were performed three times in triplicate.

### *In vivo* xenograft experiments

All the experimental protocols were approved by the Institutional Animal Care and Committee of Xinqiao Hospital, The Third Military Medical University, Chongqing, P. R. China. Four-week old male BALB/c nude mice were randomly divided into four groups, with six mice in each group. ACHN cells stably transfected with LV3-shNC, LV3-shPVT1, shPVT1+vector, and shPVT1+Mcl-1 (5 × 10^6^ cells per mouse) were injected subcutaneously into the right flanks of the mice. Tumor growth was monitored, and tumor size were measured every three days. Tumor volume was calculated using the formula of volume = (width^2^ ×length×0.5). Thirty days after injection, the mice were sacrificed and tumor weights were measured for further analysis. The primary tumors were excised, and tumor tissues were used for qRT-PCR analysis, Western blot analysis, and immunostaining analysis.

### Immunohistochemistry (IHC)

The xenograft tumors were immunostained for Ki-67, as previously described [30].

### Statistical analysis

The statistical analysis was performed using SPSS 16.0 by a blinded investigator. The data were expressed as the mean ± SD. When two groups were compared, Student's t test was used. When more than two groups were compared, one-way ANOVA followed by Tukey's Test was carried out. Monte Carlo simulation was employed to compare the expression between the tumor and non-tumor tissues. The Wilcoxon's Sign Rank Test was used to compare the expression difference among histological grades and pathological stages. The Long Rank Test analyzed the overall survival difference between higher and lower expression groups. A p-value of < 0.05 was statistically significant

## SUPPLEMENTARY MATERIALS FIGURES


