# Supplementary material for: Long noncoding RNA PVT1 inhibits renal cancer cell apoptosis by up-regulating Mcl-1
Source: Oncotarget. 2017 Oct 9;8(60):101865–75. doi: 10.18632/oncotarget.21706 (PMC5731919; doi:10.18632/oncotarget.21706)
Supplement: Supplementary file 1 [file oncotarget-08-101865-s001.pdf]

Long noncoding RNA PVT1 inhibits renal cancer cell apoptosis by up-regulating Mcl-1

SUPPLEMENTARY MATERIALS

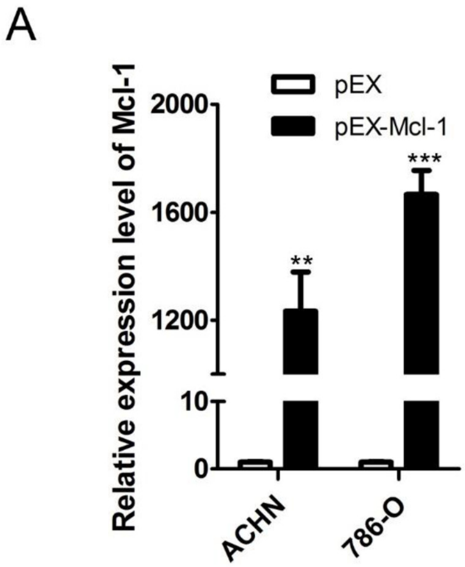

Supplementary Figure 1: qRT-PCR analysis of Mcl-1 in renal cancer cells transfected with pEX or pEX-Mcl-1. Mcl-1 expression in 786-O and ACHN cells transfected with pEX-2 or pEX-Mcl-1 were detected by qRT-PCR. ($^{**}p < 0.01$, $^{***}p < 0.001$).

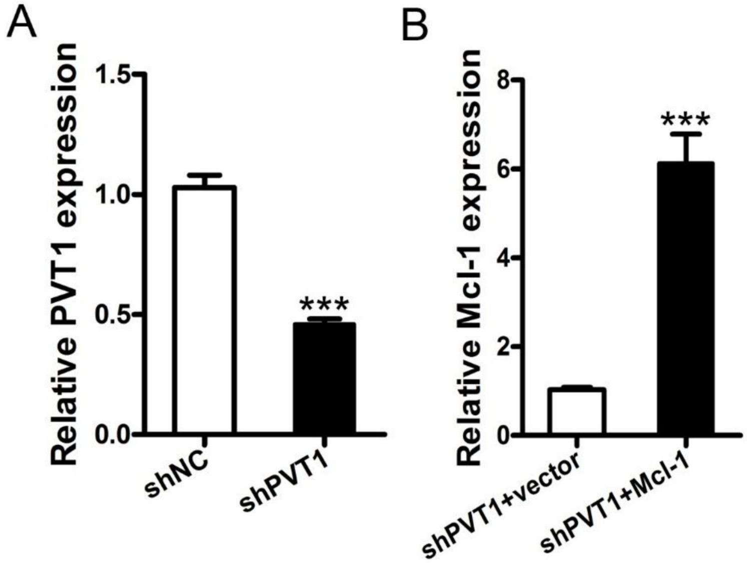

Supplementary Figure 2: qRT-PCR analysis of PVT1 and Mcl-1 in xenograft tumors. **(A)** PVT-1 expression in shNC and shPVT1 xenograft tumors. **(B)** Mcl-1 expression in shPVT1+vector and shPVT1+Mcl-1 xenograft tumors. ($^{***}p < 0.001$).

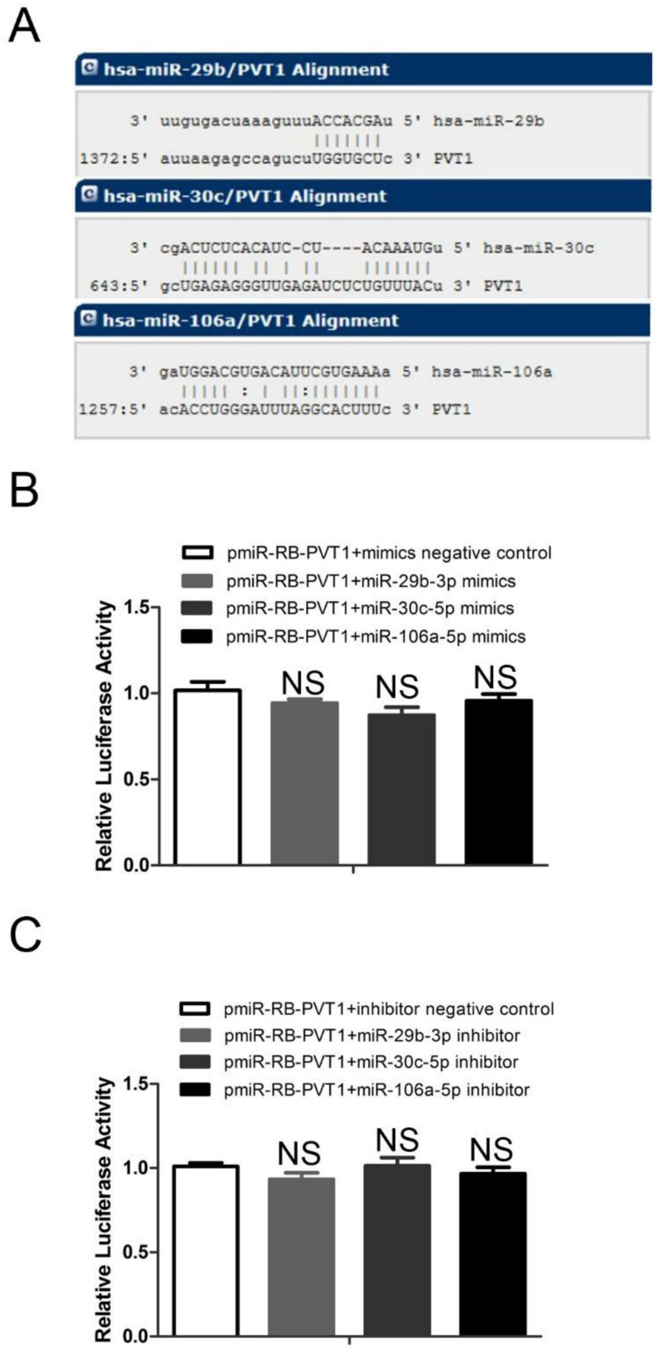

Supplementary Figure 3: There is no direct binding between PVT-1 and miR-29b-3p/ miR-30C-5p/ miR-106a-5p. (A) The putative binding sites of miR-29b-3p/ miR-30C-5p/ miR-106a-5p in PVT1. **(B)** After cotransfection withPmiR-RB-PVT1andmiR-29b-3p/ miR-30C-5p/ miR-106a-5p/miR-NC mimics for 48 h in ACHN, the luciferase activity was assayed using the Dual-Luciferase Reporter System, and normalized to the control. **(C)** The luciferase activity of PmiR-RB-PVT1 in ACHN cells cotransfected with PmiR-RB-PVT1andmiR-29b-3p/miR-30C-5p/ miR-106a-5p/miR-NC inhibitors for 48 h.
